# Blocking XIAP:CASP7-p19 selectively induces apoptosis of CASP3/DR malignancies by a novel reversible small molecule

**DOI:** 10.1038/s41419-025-07774-y

**Published:** 2025-06-18

**Authors:** Shih-Hsun Chen, Szu-Ying Wu, Yun-Xun Chang, En-Ning Lui, Chih-Kang Chang, Sheng-Wei Lin, Michael Hsiao, Jinn-Moon Yang, Po-Huang Liang

**Affiliations:** 1 Institute of Biochemical Sciences, National Taiwan University, Taipei, Taiwan; 2 Institute of Biological Chemistry, Academia Sinica, Taipei, Taiwan; 3 Genomics Research Center, Academia Sinica, Taipei, Taiwan; 4 Department of Biological Science and Technology, National Yang Ming Chiao Tung University, Hsinchu, Taiwan

**Keywords:** Apoptosis, Cancer therapy

## Abstract

X-linked inhibitor of apoptosis (XIAP) inhibits caspases 3, 7, and 9, thereby preventing cell apoptosis. Endogenous Second mitochondria-derived activator of caspase (Smac) competes out the binding of caspases with XIAP and causes apoptosis, so that Smac mimetics are under clinical trials for anti-cancer chemotherapy. We demonstrated by selectively alkylating caspase 7 (CASP7) to release the active CASP7 for killing the drug-resistant cancer cells with accumulated XIAP:CASP7 resulted from caspase-3 down-regulation (CASP3/DR). However, finding a reversible inhibitor of the protein-protein interaction (PPI) poses a significant challenge. Here, we identified a reversible XIAP:CASP7 inhibitor, **643943**, through a multiple-mode virtual screening strategy. In vitro experiments revealed that **643943** bound to CASP7, released the linker-BIR2 domain of XIAP, and activated the caspase. Removing an essential hydroxyl group on **643943** or replacing the OH-interacting Asp93 on CASP7 caused loss of **643943** cytotoxicity, revealing the binding mode. This compound thus selectively killed MCF-7 and other CASP3/DR triple-negative breast cancer cell lines, but not the cancer and normal cell lines expressing higher levels of CASP3 in vitro and in vivo. Moreover, **643943** overcame chemoresistance via down-regulating β-catenin and its associated ABC transporters in paclitaxel-resistant MCF-7 cells. Our studies not only serve as a proof-of-concept for using XIAP:CASP7 as a drug target, but also provide the first reversible XIAP:CASP7 inhibitor for cancer therapy of CASP3/DR malignancies.

## Introduction

A variety of stimuli, including chemotherapeutic agents, irradiation, and cytokines, ultimately activate caspase-3 (CASP3), a major executioner protein of proteolytic degradation, to induce cancer cell apoptosis [1]. However, caspase-3 down-regulation (CASP3/DR) in tumors is associated with chemoresistance and is correlated with poor prognosis in cancer patients with solid tumors [2–12] and leukemia [13]. Several breast cancer cell lines express significantly lower or no CASP3, while normal tissues have higher CASP3 levels [2]. For example, MCF-7 cells lack CASP3 expression as a result of a functional deletion mutation in the *CASP3* gene. They are relatively insensitive to cisplatin, doxorubicin, and etoposide, but upon reconstituting CASP3, the sensitivity of MCF-7 cells to these drugs could be recovered, suggesting that the loss of CASP3 expression may represent an important cell survival mechanism in the breast cancer patients [2]. More importantly, a significant majority of patient breast tumors lack CASP3 expression, which represents an obstacle to cancer therapy.

The cancer cells of CASP3/DR to escape from apoptosis often up-regulate the structurally and functionally similar caspase-7 (CASP7) to achieve cellular homeostasis, which can then be inhibited by X-linked inhibitor of apoptosis protein (XIAP) via protein-protein interaction (PPI), leading to accumulation of the XIAP:CASP7 complex [14]. As reported previously, we identified the Cys246, a non-active site residue of CASP7, to be alkylated by a small molecule I-Lys containing an iodomethyl ketone warhead to block the XIAP:CASP7 PPI. This disruption releases active CASP7, a 19-kDa/12-kDa heterodimer (p19/p12-CASP7), to kill the CASP3/DR cancer cells [15]. Because CASP3/DR combined with XIAP:CASP7 accumulation correlates with the aggressive evolution of clinical malignancies and a poor prognosis in cancer patients, targeting XIAP:CASP7 represents an effective treatment against these malignant cancers [15]. The strategy is safe due to the absence of XIAP:CASP7 in normal cells, which mainly express CASP3. We further showed that targeting this PPI effectively kills cancer cells with multidrug resistance resulting from miR-let-7a1-mediated CASP3/DR and re-sensitizes the cancer cells to chemotherapy-induced apoptosis [15].

XIAP, a member of the Inhibitor of Apoptosis (IAP) proteins, directly binds and inhibits upstream CASP9 and downstream CASP3/7 in the intrinsic apoptotic pathway [16]. Due to their anti-apoptotic effect, IAP proteins have been proposed for therapeutic intervention in cancers [17]. XIAP consists of three baculovirus IAP repeat (BIR) domains (BIR1, BIR2, and BIR3) and a C-terminal RING domain [18]. Although there is significant homology among the BIR domains across the IAP family, they possess different specificities for inhibiting CASP3, 7, and 9 [18]. The active sites of CASP3/7 can be occupied by the linker prior to BIR2, while their four N-terminal residues are bound to the grooves of the BIR2 domain to enhance binding affinity [19–22]. In contrast, the BIR3 domain is responsible for binding CASP9 to prevent its dimerization and activation [23, 24]. Second mitochondria-derived activator of caspase (SMAC), an endogenous IAP antagonist [25, 26], primarily associates with the BIR3 domain through its N-terminal IAP-binding motif, AVPI, to activate CASP9 [27, 28]. It also binds to the BIR2 domain to release CASP3/7 [29]. Because XIAP overexpression correlates strongly with cancer progression and chemoresistance, small-molecule XIAP antagonists [30–32] and SMAC mimetics [33–35] have been developed to inhibit the interaction between XIAP and these caspases to kill malignant tumor cells. However, due to a lack of selectivity between CASP3 and 7, some of these agents are toxic to the hematopoietic progenitor/stem cells [36] when the agents activate CASP3 from the XIAP:CASP3 that prevents apoptosis in normal proliferating cells [37]. In contrast, I-Lys only disrupts the XIAP:CASP7 by specifically targeting CASP7 without affecting CASP3 [15].

Generally speaking, a reversible inhibitor is preferable to a covalent inhibitor as a drug, but the larger interfaces of the targeted PPIs pose a great challenge toward designing small reversible inhibitors. There have only been a few successful cases of discovering small reversible inhibitors that target hot spots at the interfaces of PPIs [38]. After identifying Cys246 of CASP7 as a hot spot to disrupt XIAP:CASP7, our current study, as shown below utilized computer virtual screening to identify a small molecule that we postulated to bind reversibly to an allosteric site on CASP7 and inhibited the PPI. Computer modeling further suggested the binding mode and rationalized the disruption of the complex by the reversible inhibitor. This compound was demonstrated to be effective against a number of CASP3/DR cancers in vitro and in vivo. We report herein a novel strategy for treating CASP3/DR malignancies and the first small reversible PPI inhibitor of XIAP:CASP7.

## Results

### Using multiple-mode screening strategy to identify a reversible XIAP:CASP7 PPI inhibitor

**Fig. 1:**
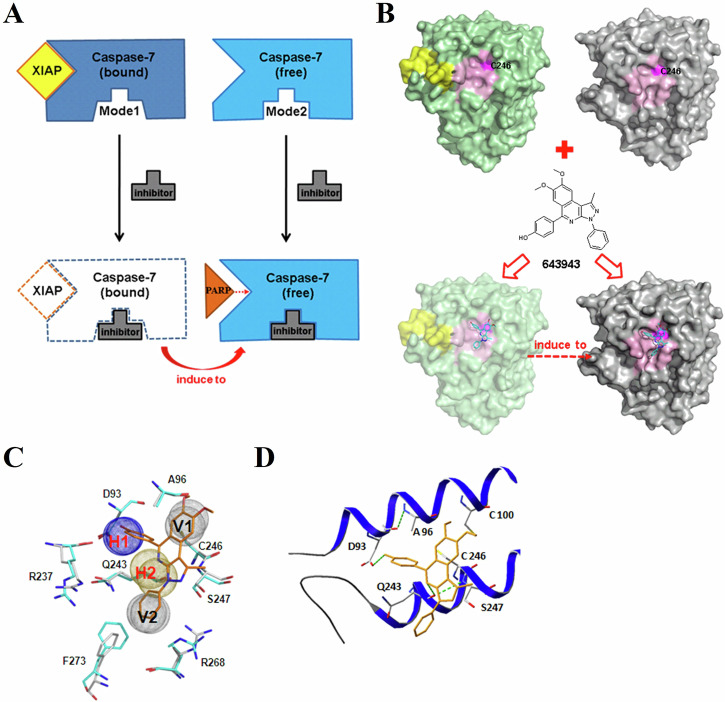
Multiple-mode screening strategy to identify the XIAP:CASP7 PPI inhibitor. **A** The cartoon binding model of a small-molecule inhibitor (gray) targeting multiple modes to block the interaction of linker-BIR2 of XIAP (yellow) with CASP7 (blue and light blue) and cause cell apoptosis. **B** The allosteric binding site, including the exposed Cys246 for I-Lys binding, can be found in both XIAP-bound and XIAP-free form CASP7 structures (PDB codes 1I51 and 1K86). The inhibitor, **643943**, binds into modes 1 and 2 to disrupt interactions between CASP7 and XIAP though inducing XIAP-bound to unbound structure and directly stabilizes active-form CASP7. **C** Multiple-mode strategy increases the chance of **643943** (orange) fitting to the binding site in CASP7. The common site-moiety map was identified by superimposing site-moiety-maps of mode 1 and mode 2 and consists of four core anchors (H1, H2, V1 and V2). **D** The docked conformation of **643943** at allosteric inhibition site of CASP7. **643943** (orange) interacts with two residues, D93 (anchor H1) and Q243 (anchor H2), through hydrogen bonds (green dashed lines).

Based on the crystal structures of the linke-BIR2-bound (PDB code 1I51 [39]) and free (PDB code 1K86 [40]) CASP7 from the Protein Data Bank [41], Cys246 is on a flat and non-contiguous surface, increasing the challenge for discovering a small-molecule reversible inhibitor of this PPI. To maximize the probability of finding a compound to bind with both forms of CASP7, we employed a two-mode screening strategy as shown in Fig. 1A. The major steps of using a two-mode screening strategy to identify the PPI inhibitor by combining site-moiety maps [42] and rank-based consensus (RCS) scoring method [43] are outlined in Supplementary Fig. S1. For each binding mode, we used GEMDOCK [44] to screen compounds docked into the site of C246 (Supplementary Fig. S1A) and the nearby residues D93, A96, and Q243 (Supplementary Fig. S1B). Other residues within 6 Å of these four residues were selected as the binding environment for a virtual screening using more than 50,000 Sigma-Aldrich compounds (Supplementary Fig. S1C). The top 10% ranked compounds were selected based on the piecewise linear potential (PLP) score [44] for generating residue-compound interacting profiles (Supplementary Fig. S1D). After the profiles were generated, we inferred the site-moiety maps of mode 1 (with linker-BIR2 bound, shown in Supplementary Fig. S1E) and mode 2 (without any ligand bound, shown in Supplementary Fig. S1F) by determining statistically significant interacting residues and moieties. In order to increase the preference of binding environment for inhibitors and enhance the efficiency of blocking the XIAP:CASP7 complex, we proposed that the PPI inhibitors would bind to the two modes by fitting common site-moiety-map with consensus anchors (Supplementary Fig. S1G). Finally, we selected compound candidates that were top ranked in multiple binding modes based on the RCS method combining the PLP and anchor scores on multiple modes for our bioassay (Supplementary Fig. S1H).

The multiple-mode strategy allowed us to identify a compound **643943** (structure shown in Fig. 1B) that fits into the Cys246 region of CASP7. Upon the hit binding, the linker-BIR2-bound CASP7 structure should be converted to a free-form CASP7 structure. It should be noted that Cys246 is located approximately 18 Å away from the catalytic site Cys186 in the PPI interface. Hence, **643943** binding to Cys246 in an allosteric site did not interfere with the CASP7 activity, but just caused dissociation of XIAP to activate CASP7. Although the site-moiety maps of the two binding modes for **643943** are not the same (Supplementary Fig. S1E, F), the four core residues (D93, A96, Q243, and C246) consist of two electrostatic force/H-bond anchors (H1 and H2) and two van der Waals (vdW) anchors (V1 and V2) (Fig. 1C and Supplementary Fig. S1G).

For each anchor, we identified a set of chemically related entities (Supplementary Fig. S2), supporting the concept that conserved binding residues lend a given hot spot a unique chemical-physical binding environment. Among the top-ranking 1000 compounds, 553 small molecules form electrostatic force/hydrogen bonds with residues K92, D93, and R237 in anchor H1; 864 form vdW interactions with residues Q243, R268, and F273 in the V2 anchor (Supplementary Fig. S2). Nitro, ketone, enol, and amide functional groups are the major chemical moieties of the H1 anchor (Supplementary Fig. S2). Six-membered rings, simple aromatic rings, alkene, and phenol are the major functional groups for vdW anchors V1 and V2. An anchor in a site-moiety map can define the interacting preference between a binding pocket (formed by several residues) and its preferred moieties. For example, for binding mode 2, anchor H1 possesses a pocket with three residues (K92, D93, and R237) that often form electrostatic force/hydrogen bonds with three main moiety types (i.e. nitro, amide, and ketone). H2 anchor is located at the hydrogen-bonding environment made up of Q243 and S247. The V1 anchor consists of a pocket with conserved interactions between residues A96, C246, and E250 with bulky moieties, such as aromatic ring and heterocyclic group. The V2 anchor bears a vdW-bonding environment with residues Q243, R268, and F273.

Finally, the identified **643943** highly agrees to the common site-moiety map (Fig. 1C) and fits well into the allosteric inhibition pocket of CASP7. The hydroxyl group of **643943** may form a hydrogen bond with Asp93, and the backbone of Q243 may contact with the nitrogen atom of **643943** via a hydrogen bond as well (Fig. 1D).

### 643943 disrupts XIAP:CASP7 complexes and induces CASP7-mediated apoptosis

**Fig. 2:**
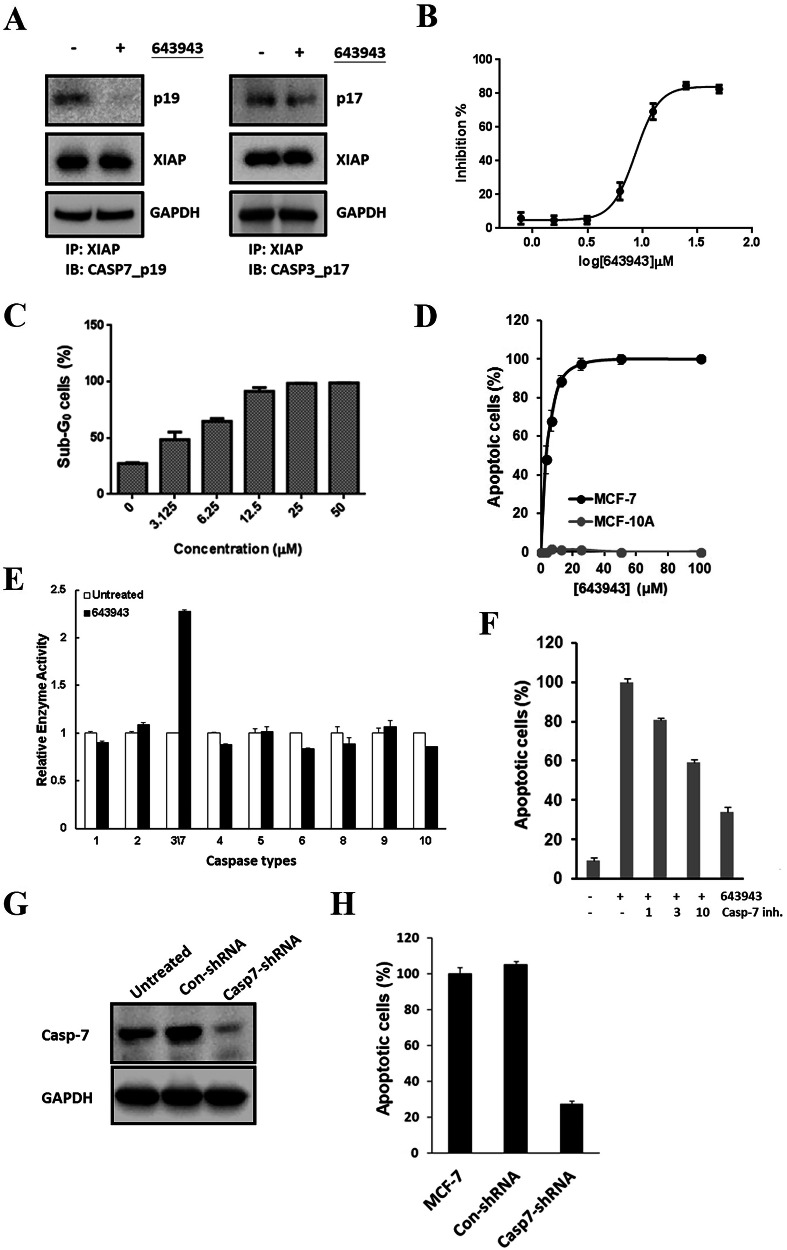
643943 triggers CASP7-mediated apoptotic signaling by disrupting the XIAP:CASP7 complex. **A** Immunoprecipitation/Western blot analysis of XIAP:CASP7 complex in MCF-7 cells and XIAP:CASP3 complex in MCF-10A treated with **643943** (20 μM) for 4 h, respectively. **643943** efficiently blocked the PPI between XIAP and CASP7. GAPDH was used as a loading control. **B** Cytocidal effects of **643943** on MCF-7 cells based on MTT assay. Cells were treated with **643943** at the indicated concentrations for 24 h and live cells were determined by MTT assay. **C** Cytocidal effects of **643943** on MCF-7 cells based on sub-G_0_ arrest. Cells were treated with **643943** at the indicated concentrations for 24 h. Accumulation of cells in the sub-G_0_ fraction was determined by PI-based flow cytometric analysis. **D** Cytocidal effects of normal MCF-10A cells. Cells were treated with **643943** at the indicated concentrations for 24 h. Accumulation of cells in the sub-G_0_ fraction was determined by PI-based flow cytometric analysis. **E** Determination of intracellular caspase activities in untreated (white bars) or **643943**-treated (black bars) MCF-7 cells for 24 h. **F** Relative apoptosis percentages of MCF-7 cells treated with 20 μM **643943** for 24 h in the absence or presence of the CASP7 inhibitor (MPS) at the indicated doses. **G** Immunoblotting for CASP7 and GAPDH (control for protein loading) and **H** relative apoptotic percentages of the MCF-7 cells and the cells transfected with control shRNA (Con-shRNA) or specific shRNA (CASP7-shRNA). **A**–**H** Data were obtained from three independent experiments.

To test if **643943** could disrupt XIAP:CASP7 complexes and trigger CASP7-mediated apoptosis, we used MCF-7 that expresses CASP7 but not CASP3 as a model system as previously described [15]. As shown in the left panel of Fig. 2A, immunoprecipitation (IP) analysis using the XIAP antibody to pull down the complex, followed by Western blot detecting the level of CASP7, revealed that **643943** reduced the level of the intracellular XIAP:CASP7 complex within 4 h of treatment. In contrast, **643943** failed to disrupt the PPI of CASP3 with XIAP in the normal MCF-10A cells, which express CASP3 rather than CASP7 (Fig. 2A right panel). Among the tested candidates, **643943** exhibited the greatest cytotoxic effect on MCF-7 cells, with an EC_50_ of 8.48 ± 0.06 μM (Fig. 2B) in the MTT assay, consistent with flow cytometry analysis of the sub-G_0_ cell population (Fig. 2C). However, **643943** treatment did not kill the normal breast MCF-10A cells at 100 μM (Fig. 2D). To further confirm that its cytotoxic effect proceeded through apoptotic signaling, we determined the activation state of all caspases involved in the extrinsic and intrinsic apoptotic pathways in MCF-7 cells. Only CASP7 activity was detected in MCF-7 cells after treatment with **643943** for 24 h (Fig. 2E). Moreover, pharmaceutical inhibition of the CASP7 signaling axis by its specific inhibitor, (S)-(+)-5-[1-(2-methoxymethylpyrrolidinyl)sulfonyl]isatin (MPS), suppressed **643943**-induced apoptosis in MCF-7 cells in a dose-dependent manner (Fig. 2F). When CASP7 was depleted via shRNA (Fig. 2G), the **643943**-induced apoptosis in MCF-7 cells was also reduced (Fig. 2H). These results clearly demonstrate that removal of XIAP inhibition by **643943** is a crucial process for eliciting CASP7-mediated apoptotic signaling cascades in MCF-7 cells.

### 643943 binds to CASP7 and dissociates it from the complex

**Fig. 3:**
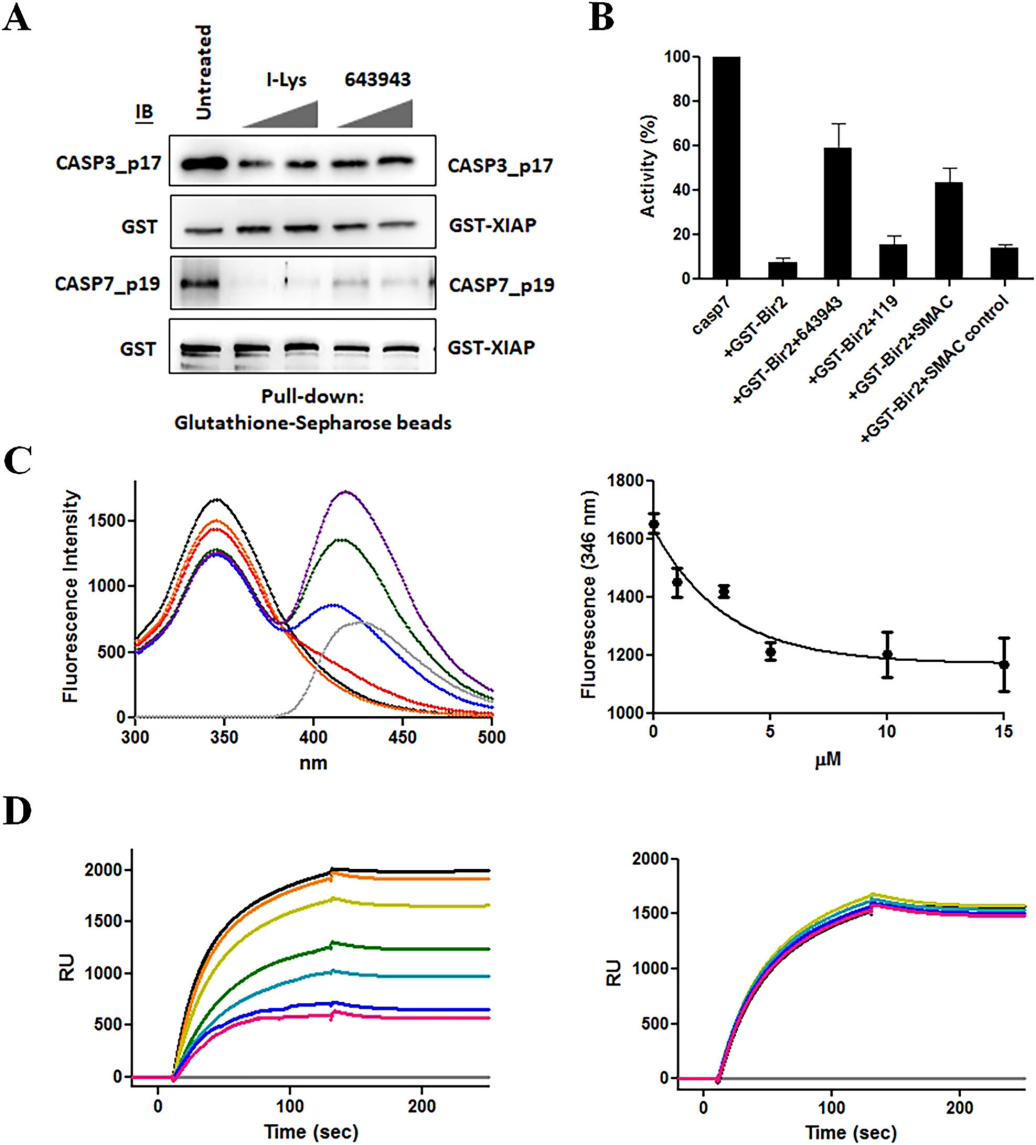
643943 releases CASP7 from the inactive complex with XIAP. **A** In vitro binding assay for GST-XIAP interaction with CASP7 in the presence of increasing **643943** concentrations (0.1 and 0.5 mM). GST-XIAP was pulled down using Glutathione beads and CASP7 was detected via Western blot. Like I-Lys, **643943** disrupted the GST-XIAP:CASP7, but not GST-XIAP:CASP3 complexes. **B** Activity levels of CASP7 complexed with GST-linker-BIR2 and subsequently treated with 100 μM of different agents including **643943,** **119**, a peptide AVPFVASLPN derived from SMAC, and a mutant peptide. **C** Fluorescence spectra of CASP7 in the absence (black) and presence of different concentrations (1, 3, 5, 10, and 15 μM) of **643943** as shown in orange, red, blue, green, and purple, respectively (left panel). The fluorescence spectrum (gray) of **643943** has a *λ*_max_ of 420 nm, so that the addition of **643943** did not contribute to the fluorescence changes at 346 nm, the *λ*_max_ of CASP7. The right panel shows the changes of fluorescence at 346 nm during titration. **D** BIAcore traces showing the binding of 0.28 μM GST-linker-BIR2 in the absence (black) and presence of different concentrations of **643943** as shown in orange, yellow, green, cyan, blue, and pink, respectively (left panel) and **119** as a negative control (right panel). The concentrations of **643943** and **119** used were 0, 0.5, 10, 15, 20, 30, and 40 µM. **A**–**D** Data were obtained from three independent experiments.

To further confirm that **643943** can directly bind to CASP7 and release it from the inhibitory complex, we prepared recombinant CASP7 and GST-XIAP for in vitro experiments. As shown in Fig. 3A, using Glutathione-beads to pull down GST-XIAP and detecting the associated CASP7 level with Western blot, **643943** and I-Lys (positive control) dramatically disrupted the XIAP:CASP7 complex. Moreover, GST-linker-BIR2 inhibited CASP7 activity, whereas **643943** increased the CASP7 activity from the complex. The response to **643943** was similar to the activation of CASP7 by a previously reported peptide, AVPFVASLPN, derived from Smac [45], in contrast to the lack of response to the negative control peptide and **119**, a **643943** analog lacking the Asp93-interacting OH group (Fig. 3B).

Next, we titrated **643943** into the solution containing CASP7 and measured the fluorescence spectral changes. As shown in the left panel of Fig. 3C, the addition of **643943** changed the spectra of CASP7 in a dose-dependent manner, causing the decrease of fluorescence intensities at 346 nm (the *λ*_max_ of free CASP7) and the concurrent increase at 420 nm (the *λ*_max_ of **643943**), by forming increasing levels of **643943**-bound CASP7. In contrast, **119** (**643943** without an OH group) failed to change the spectra (data not shown), indicating no binding.

Furthermore, we coated the recombinant His-tagged CASP7 on a NiNTA-chip and performed BIAcore experiments to detect the change in SPR signal during the binding of **643943** with CASP7. Upon injection, **643943** gave concentration-dependent binding signals with the immobilized CASP7 and the calculated *K*_d_ was 3.1 μM (data not shown), similar to the EC_50_ of **643943** in killing MCF-7. In the absence and presence of different concentrations of **643943** co-injected with a fixed concentration of GST-linker-BIR2 into the chip, we observed a dose-dependent inhibition of the GST-linker-BIR2:CASP7 complex formation (Fig. 3D, left panel). In contrast, **119** failed to interfere with the binding of GST-linker-BIR2 with CASP7 (Fig. 3D, right panel). Overall, our in vitro studies confirmed that **643943** could bind to CASP7 and compete with the binding of linker-BIR2 with CASP7.

### Computer modeling showing the mechanism of XIAP:CASP7 complex disruption by the reversible PPI Inhibitor

**Fig. 4:**
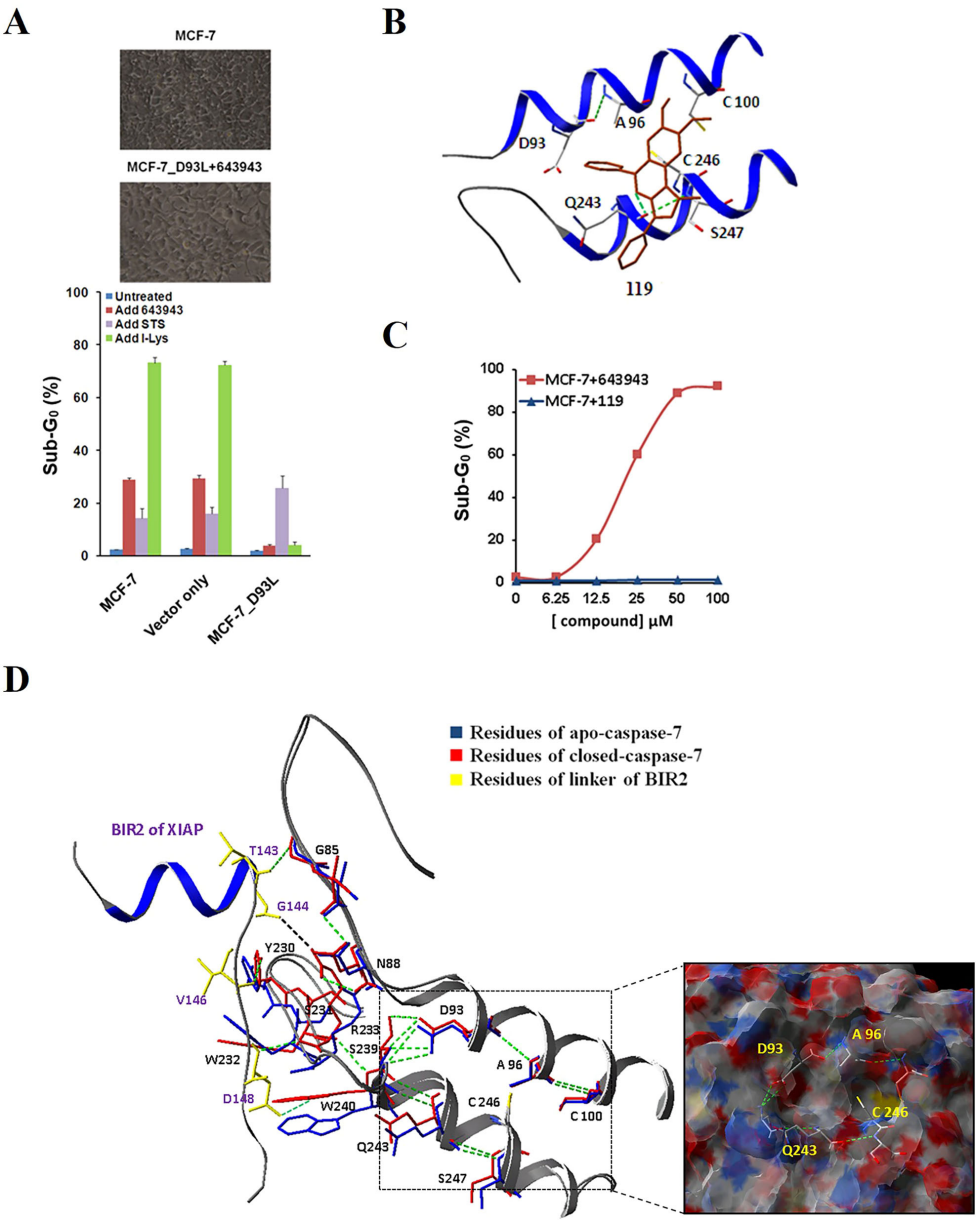
Allosteric mechanism of 643943 disrupting XIAP:CASP7. **A** Sub-G_0_ cell populations of MCF-7, MCF-7 transfected with vector only, and MCF-7 variant cells stably expressing CASP7 with D93L mutation (MCF7_D93L) after treatment with I-Lys (1 μM), STS (1 μM), and **643943** (20 μM) for 24 h. **B** The binding mode of **119**, an analog of **643943** without the D93 interacting OH group. **C** Sub-G_0_ cell populations of MCF-7 cells treated with **643943** and **119** measured by flow cytometry. The results showed no **119** induced-sub-G_0_ accumulation in MCF-7 cells, suggesting D93 is a key interacting residue for XIAP:CASP7 PPI. **D** Hydrogen bound interaction networks among significant residues of XIAP:CASP7 interface and four core residues (D93, A96, Q243, and C246) of the allosteric site are shown. D93 is indirectly (via S239) linked to three interacting residues in the linker preceding BIR2, especially D148.

Because computer docking revealed that the interaction of an OH group in **643943** with Asp93 of CASP7 is important for binding, we established an MCF-7 cell line that can stably overexpress mutant CASP7_D93L. Overexpression of this mutant CASP7 significantly decreased the **643943**-induced sub-G_0_ cell population, indicating the importance of Asp93 for binding. Efficacy of the control, an apoptotic agent staurosporine (STS), was not affected by the mutation (Fig. 4A). Compound **119**, which lacks the OH group and is therefore unable to form an H-bond with Asp93 (Fig. 4B), failed to kill the cells (Fig. 4C). These results suggest that **643943** binds with CASP7 through Asp93 and activates CASP7 from XIAP:CASP7, thereby inducing cell death.

To investigate how the interaction between **643943** with Asp93 can lead to the dissociation of XIAP from CASP7, we analyzed the hydrogen-bond network based on the crystal structure of linker-BIR2-bound CASP7. As shown in Fig. 4D, G144, V146, and D148 of the linker (shown in yellow) form hydrogen bonds with the residues R233, Y230, and W240 (shown in red) of CASP7. D148 of the linker, which has been suggested as a key residue for the interaction [20, 46], is bound via a hydrogen bond with W240 of CASP7. W240 is indirectly (via S239) connected to D93. Therefore, when D93 is occupied by **643943**, those interactions between XIAP and CASP7 are broken. Moreover, **643943** broke the H-bond between S239 and D93 and affected two other interactions between XIAP and CASP7 through R233, which directly binds with G144 of the linker and indirectly links to Y230 via S231.

### 643943 exhibits selective lethality in XIAP:CASP7 accumulated breast cancer cell lines and anti-tumor activity in vivo

**Fig. 5:**
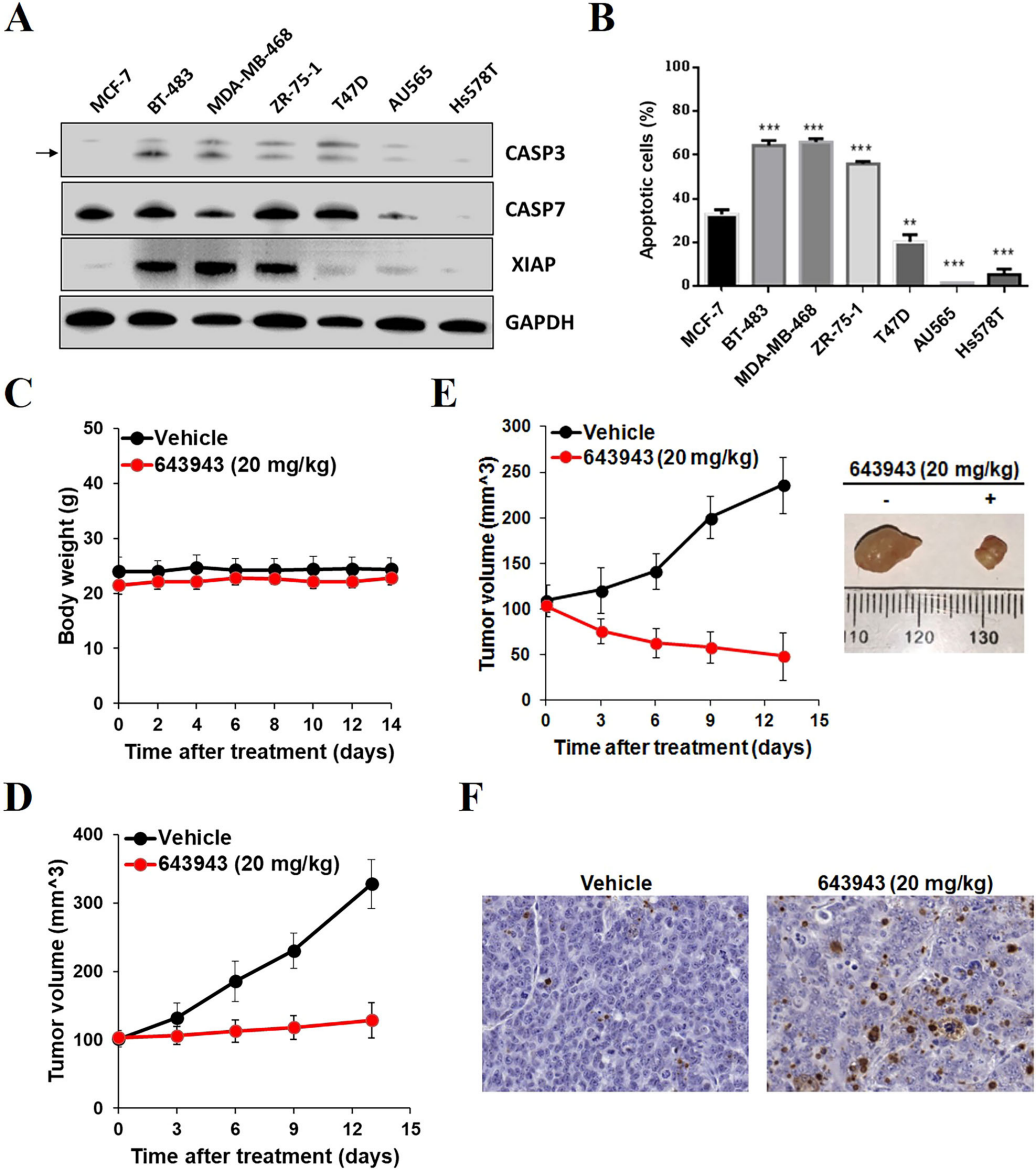
643943 suppresses CASP3/DR cancer cells and exhibits anti-tumor activity in vivo. **A**, **B** Western blot analysis and apoptotic cell populations of a panel of breast cancer cell lines treated with 20 μM **643943** for 24 h. The CASP3, CASP7, and XIAP expression levels of these cancer cells were analyzed by Western blot using GADPH as a loading control. Data were obtained from three independent experiments. ** and *** denote *p* < 0.01 and *p* < 0.001, respectively. **C** Body weight of mice. Mice were dosed intraperitoneally with 30% solutol (vehicle, *n* = 3) or **643943** in 30% solutol at 20 mg/kg (*n* = 3) once daily for 14 days. Body weights of mice were measured and presented as mean ± SD. **D**, **E** Tumor growth of MCF-7 and MDA-MB-468 cells post-treatment without or with **643943**. Mice were implanted with MCF-7 (**D**) or MDA-MB-468 (**E**) cells and treated without (vehicle, 30% solutol, *n* = 6) or with **643943** at 20 mg/kg/day (*n* = 6) for 2 weeks. Tumor volumes were measured every 3 days and presented as mean ± SD. **F** Analysis of apoptosis, detected by TUNEL. Shown are immunohistochemistry images from representative sections of tumor samples from **643943** and vehicle-treated mice.

Next, we examined the selectivity of **643943** in killing a panel of breast cancer cell lines that express CASP3 at different levels, particularly those reported CASP3/DR. We examined their CASP3, CASP7, and XIAP expression levels via Western blot analysis. Our data showed that those cancer cell lines with higher levels of XIAP:CASP7 (e.g. MCF-7, BT-483, MDA-MB-468, and ZR-75-1) were hypersensitive to **643943** (Fig. 5A, B), consistent with that **643943** disrupts the XIAP:CASP7 complexes. We further measured the EC_50_ values of **643943** against BT-483, MDA-MB-468 and ZR-75-1 to be 4.1, 5.2, and 6.7 μM, respectively, according to MTT and flow cytometry assays (Supplementary Fig. S3). As expected, XIAP:CASP7-proficient tumor cells have an increased dependence on **643943**-mediated effects (Fig. 2B and Supplementary Fig. S3).

We then sought to test if **643943** is able to treat tumors in vivo. To examine the toxicity of **643943** in mice, 6-week-old immunodeficient mice (NOD-scid) were intraperitoneally administered with **643943** in 30% solutol at 20 mg/kg (*n* = 3) daily for 2 weeks. Mice were weighed per 2 days and observed for any adverse effects. Compared to vehicle control (30% solutol, *n* = 3), no significant loss in weight (Fig. 5C) and signs of ill effects were observed in mice treated with **643943**, suggesting that this small molecule is non-toxic to mice at 20 mg/kg and can be used for further mouse model testing. To evaluate the anti-cancer effect of **643943** in vivo, MCF-7- and MDA-MB-468-bearing mouse models were established. With vehicle group as control, the administration of **643943** by intraperitoneal injections at 20 mg/kg led to a significant reduced tumor volume (Fig. 5D, E) and induced an increase for apoptosis determined by TUNEL analysis (Fig. 5F). These results demonstrated that **643943** has the therapeutic potential for CASP3/DR or XIAP:CASP7 accumulated cancers.

### 643943 enhanced efficacy of chemotherapeutic drugs and overcame chemoresistance in CASP3/DR cancer cells

**Fig. 6:**
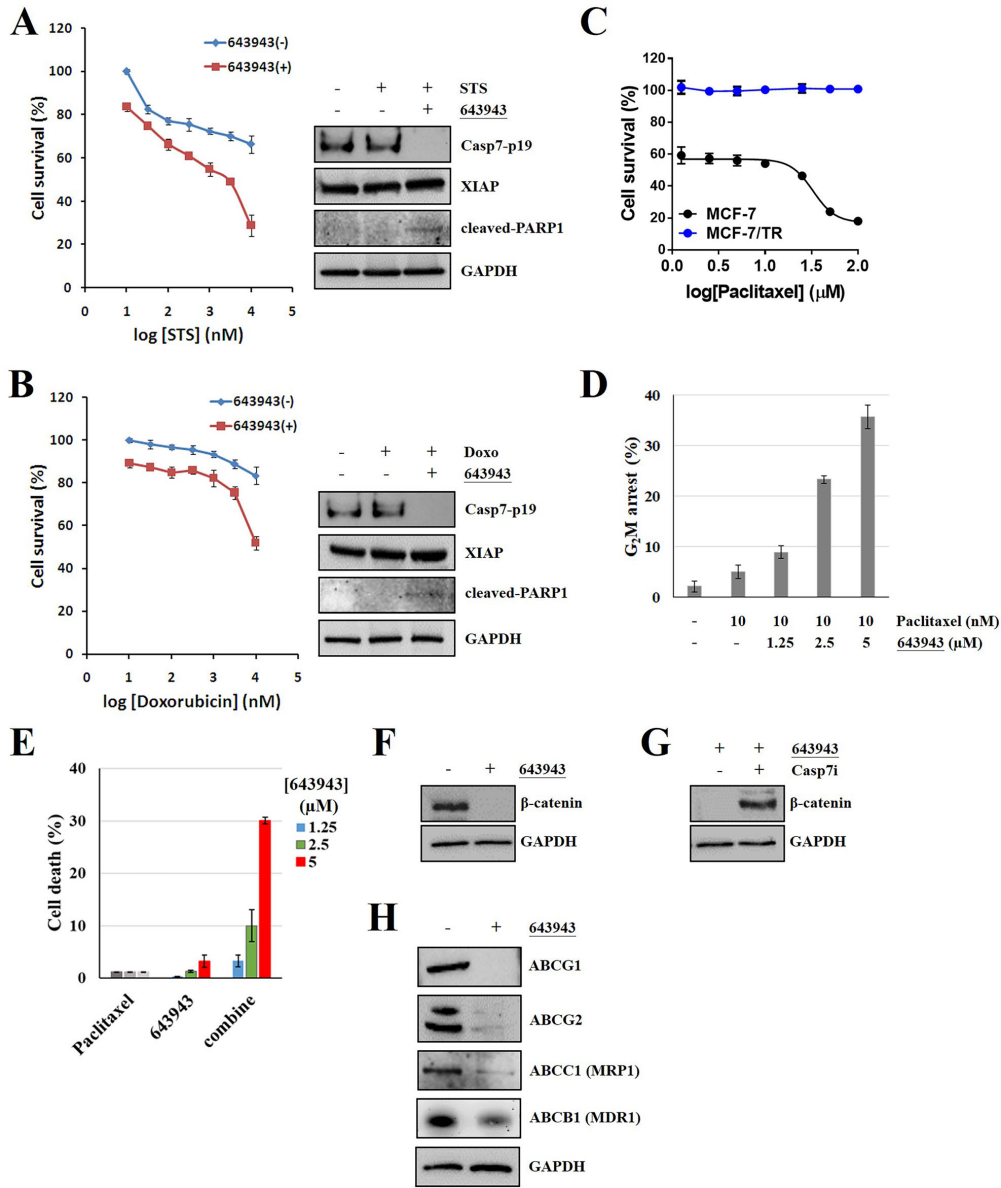
643943 exhibits synergistic effect with anti-cancer drugs and effectively sensitizes chemoresistant cancer cells to chemotherapy. **A**, **B** Combination treatments with **643943** and anti-cancer drugs (STS and doxorubicin) exhibit synergistic effect in MCF-7 cells. Immunoprecipitation/Western blot analysis of XIAP:CASP7-p19 complex and cleaved PARP1 in MCF-7 cells treated without or with indicated compounds. GAPDH was used as a loading control. **C** Cell survival analysis following paclitaxel treatment. MCF-7 and paclitaxel-resistant MCF-7 (MCF-7/TR) cells were treated with paclitaxel at the indicated concentrations for 48 h. **D** G_2_M arrest analysis following combination treatment of **643943** and paclitaxel. **643943** treatment significantly recovers the efficacy of paclitaxel in MCF-7/TR. **E** MTT assays of MCF-7/TR cells treated with **643943**, paclitaxel or combination treatment of **643943** and paclitaxel. Cell death of MCF-7/TR cells were analyzed following treatments of 10 nM paclitaxel, **643943** at the indicated concentrations or combination for 24 h. **F**–**H** Western blot analysis of β-catenin, ABC transporters, and GAPDH using their specific antibodies in MCF-7/TR treated with DMSO, 5 µM **643943** or 10 µM CASP7 inhibitor (MPS). GAPDH was used as a loading control. **A**–**H** Data were obtained from three independent experiments.

Because the formation of XIAP :p19/p12-CASP7 impairs STS-induced apoptosis in MCF-7 cells, we wondered if blocking XIAP:p19/p12-CASP7 could enhance the efficacy of STS in CASP3/DR cancer cells. Interestingly, combining STS or doxorubicin treatment with **643943** significantly enhanced their cytotoxic effectiveness in MCF-7 cells (Fig. 6A, B). This synergistic effect is most likely due to **643943** disrupted the PPI between XIAP and the active form of p19/p12-CASP7 during STS- and doxorubicin-induced apoptosis in MCF-7 cells.

Moreover, according to our previous study [15], we found that CASP3/DR is highly related to chemoresistance mediated by *MIRLET7A1* (a microRNA against *CASP3*) in breast cancer cells. Preventing XIAP from binding to the emerging p19/p12-CASP7 upon apoptotic proteolysis of pro-CASP7 can assist chemotherapy against malignancies with CASP3/DR. To verify whether **643943** can be an adjuvant for chemoresistant breast cancer cells, we generated paclitaxel-resistant MCF-7 cells (7TR) (Fig. 6C) and attempted to induce 7TR cells death by combining **643943** with paclitaxel. Notably, **643943** treatment synergistically potentiated paclitaxel-induced G_2_M arrest in 7TR cells (Fig. 6D) and induced cell death of 7TR cells (Fig. 6E), indicating **643943** may overcome the resistant mechanism and restore the paclitaxel sensitivity in 7TR cells.

Numerous clinical data revealed that the multi-drug resistance phenotype in tumors is associated with the overexpression of ABC transporters [47]. In addition, β-catenin, an important modulator for transcription of certain ABC transporter genes, was reported to be associated with chemoresistance in multiple types of human cancer [48–50]. To figure out if our finding is associated with the β-catenin-mediated pathway, we estimated the expression level of β-catenin with or without **643943** treatment. Surprisingly, down-regulation of β-catenin was observed in the presence of **643943** compared with mock control (Fig. 6F) and was rescued by treatment with a CASP7 inhibitor (MPS) (Fig. 6G). Moreover, a decrease in certain ABC transporters levels was detected following **643943** treatment (Fig. 6H). It suggests that blocking XIAP:CASP7 is a feasible strategy for β-catenin-dependent chemoresistance. Taken together, these results suggest XIAP:CASP7 inhibitor is a highly potent adjuvant for chemotherapy against malignancies with CASP3/DR.

## Discussion

Caspase-mediated apoptosis has accounted for a major route for anti-cancer therapy. However, cancer cells sometimes escape drug toxicity by down-regulating the major apoptosis executor, CASP3. In our previous study [15], we not only found that the XIAP:CASP7 complex can serve as an effective chemotherapeutic target for treating CASP3/DR malignancies, but also identified a site for disrupting the PPI. In this study, we have developed a multiple-modes virtual screening strategy with site-moiety maps to discover a reversible inhibitor that can simultaneously bind into multiple modes of one binding site to disrupt the PPI and convert XIAP-bound to free CASP7 (Fig. 1). We successfully identified the first non-covalent and allosteric inhibitor, **643943**, for selectively disrupting XIAP:CASP7 complexes as confirmed by cell-based assays (Fig. 2) and in vitro activity and binding assays (Fig. 3). Based on computer modeling, **643943** may form the critical H-bond with Asp93 of CASP7, allowing the compound to allosterically disrupt the PPI (Fig. 4). Activation of CASP7 by **643943** thus induced apoptosis in CASP3/DR cancer cells in the cell-based and animal studies (Fig. 5). **643943** resulted in synergistic responses to anti-cancer drugs, reversing mechanisms of β-catenin-dependent chemoresistance by reducing expression levels of ABC transporters (Fig. 6). This study provides a workable method for discovering a reversible PPI inhibitor through structure-based screening, which serves as a proof-of-concept for using XIAP:CASP7 as a target and potentially leads to a new drug for treating CASP3/DR malignancies in clinic.

Finding a small and reversible PPI inhibitor represents a significant challenge due to large interface of a tight PPI. Unlike a protein-small molecule interface with a ~300–1000 Å^2^ size, such as an enzyme active site, a PPI interface has a significantly larger interface covering ~1500–3000 Å^2^, heightening the difficulty of developing a reversible PPI inhibitor. In the past, there were only a few successful cases, e.g. Tirofiban, targeting the PPI of integrins and their receptors for cardiovascular conditions [51], and Maraviroc, an anti-HIV drug that prevents the interaction of HIV-1 gp120 and CCR5, thereby blocking HIV-1 from entering T-cells [52]. There are several potent PPI inhibitors under development, such as the PPI inhibitors that disrupt the interaction between IL-2 and the α-chain of the IL-2 receptor, the PPI inhibitors that bind B-cell lymphoma 2 (Bcl-2) family members Bcl-2 and Bcl-XL to activate the pro-apoptotic molecule BAK (Bcl-2-antagonist/killer) or another pro-apoptotic molecule, BAD (Bcl-2 antagonist of cell death) for cancer therapy, the PPI inhibitors that interfere the binding of the human protein double minute 2 (HDM2) with the tumor-suppressor protein p53 to kill cancers, the PPI inhibitors that disrupt the crucial interaction of the viral transcription factor E2 and the viral helicase E1 for the viral life cycle of Human Papilloma Virus (HPV), the PPI inhibitors that disrupt the complex of FtsZ, a homolog of eukaryotic tubulin, and ZipA, a membrane-anchored protein in certain Gram(−) bacteria, and the PPI inhibitors that block the binding of cytokine tumor-necrosis factor (TNF), a key factor in inflammatory response, to its receptor for treating arthritis [38]. In spite of significant advancement in technology, such as fragment-based approaches, stapled peptides, alternatives to orthosteric inhibition, and antibody-aided technologies [53], finding small molecules to modulate PPI remains an enormous challenge. As reported here, we identified the first reversible XIAP:CASP7 PPI inhibitor by using virtual screening with input of our previously developed tools and demonstrated the compound efficacy and safety through both in vitro and in vivo experiments in CASP3/DR malignancies.

In fact, activating cellular caspases is a useful strategy for fighting cancers. There are agents developed to activate CASP3, such as PAC-1 that activates proCASP3 in vitro by sequestering inhibitory zinc ions, thus allowing proCASP3 to autoactivate to CASP3 [54] and KRN5500 that induces processing of CASP9 pro-form to active form [55]. Targeting the BIR2 and BIR3 domains of XIAP and other IAPs, there are several SMAC mimetics in clinical trials, including AT406 [56], TL32711 (birinapant) [57], LCL161 [58], AEG40826, CUDC427, and others [17] to release active CASP3 and CASP9 to kill cancers. However, the XIAP:CASP complexes in normal cells might also be targeted to release caspase 3 or 9, likely causing toxicity. Here, we have characterized **643943** to solely target CASP7, should be more selective. Therefore, our data represents a promising strategy for the treatment of CASP3/DR malignancies, and **643943** is a starting point in this series.

## Materials and methods

### Chemicals

Compounds **643943** and **119** were purchased from ChemBridge (CA, USA). CASP3/7 inhibitor, MPS, was purchased from Calbiochem (Merck KGaA, Darmstadt, Germany). Smac mimetics, STS, and paclitaxel were purchased from MilliporeSigma (Merck, USA). Doxorubicin was purchased from Cyrusbioscience (New Taipei City, Taiwan). Antibodies against CASP3 (cleaved Asp175), CASP7 (cleaved Asp198), and GAPDH were purchased from GeneTex (CA, USA). Antibodies against XIAP (for immunoprecipitation) and shRNA for CASP7 were obtained from Santa Cruz Biotechnology (TX, USA). Antibodies against CASP7, GST, and cleaved PARP were purchased from Cell Signaling (MA, USA). Antibodies against β-catenin, ABCG1, ABCG2, ABCC1 (MRP1), and ABCB1 (MDR1) were purchased from Proteintech (Hubei, P.R.C). pET-28a and pGEX-4T-1 vectors for expressing recombinant proteins were from Novagen (Merck Biosciences, Germany). pIRES2-EGFP vector was from Clontech (CA, USA).

### Cell culture

MCF-7 cells were obtained from the Bioresource Collection and Research Centre (Hsinchu, Taiwan) and cultivated in Eagle-MEM supplemented with 10% FBS. BT-483, MDA-MB-468, ZR-75-1, T47D, AU565 and Hs578T breast cancer cells were obtained from Dr. Michael Hsiao (Genomics Research Center, Academia Sinica, Taipei, Taiwan). Normal MCF-10A mammary cells were maintained in DMEM/F12 medium with 5% horse serum and supplemented with 20 ng/ml epithelium growth factor, 0.5 mg/ml hydrocortisone, 100 ng/ml cholera toxin, and 10 μg/ml insulin. BT-483 cells were cultured in RPMI-1640 medium with 20% FBS and Insulin. MDA-MB-468 cells were cultured in Leibovitz’s L-15 medium with 10% FBS. Hs578T cells were cultured in DMEM medium with 10% FBS. ZR-75-1, T47D and AU565 cells were cultured in RPMI-1640 medium with 10% FBS. MDA-MB-468 cells were used in a free gas exchange with atmospheric air. Other cell lines were cultured in an atmosphere of 5% CO_2_ at 37 °C.

### Expression and purification of CASP7 and GST-linker-BIR2

The human CASP7 cDNA sequence was cloned into the pET28a expression vector, and the XIAP linker-BIR2 domain (residue 124-240) was cloned into the pGEX-4T-1 vector containing N-terminal GST tag. The correct plasmids were confirmed by DNA sequencing. The recombinant C-terminally 6-His-tagged CAS7 and N-terminally GST-tagged linker-Bir2 were overexpressed in *E. coli* strain BL-21(DE3). A 10 ml overnight culture of a single transformant was used to inoculate 1 L of fresh LB medium containing 100 µg/ml ampicillin for CASP7 and 50 µg/m kanamycin for GST-linker-BIR2. The cells were grown at 37 °C to A600 = 0.8 and induced with 0.5 mM isopropyl-β-thiogalactopyranoside (IPTG) for 22 h at 16 °C. The cells were harvested by centrifugation at 7000 × *g* for 15 min, and the pellet was suspended in lysis buffer (25 mM Tris-HCl, 150 mM NaCl, and 10% glycerol, pH 7.5). A French-press instrument (Constant Cell Disruption System) was used to disrupt the cells at 20,000 psi and centrifuged at 20,000 × *g* for 2 h to discard the debris. For CASP7 protein, the cell-free extract was loaded onto Ni-NTA column, which was equilibrated with lysis buffer containing 5 mM imidazole. The column was washed with 10 mM imidazole followed by 30 mM imidazole-containing lysis buffer, and then protein was eluted with lysis buffer with 300 mM imidazole. For GST-linker-Bir2, the cell-free extract was loaded onto a glutathione-coated column. The column was washed with lysis buffer, and the protein was eluted with lysis buffer containing 10 mM glutathione. The eluted proteins were dialyzed against lysis buffer to removed imidazole or reduce-glutathione. The protein concentrations were determined by the BCA protein assay kit (BioRad, USA) with BSA as standard. SDS-PAGE analysis was used to ensure the purity of the proteins.

### Construction of expressing plasmids and the mutant stably expressing cell line

Human CASP7 (NM:033339.3) cDNA was amplified from human cDNA library (Invitrogen, CA, USA) by sticky-end PCR procedure using forward/backward primers, 5’-gcgttcgaaacggaacagacaaactagccgaggcgctc-3’/5’-gagcgcctcggctagtttgtctgttccgtttcgaacgc-3’ and cloned into pIRES2-EGFP vector. The constructed plasmid DNAs were used as template for site-directed mutagenesis. The generation of CASP7 D93L mutant was carried out by using QuikChange® Site-Directed Mutagenesis Kit (Strategene, TX, USA). To generate MCF-7 variant stably expressing CASP7 D93L mutant, MCF-7 cells were transfected with each plasmid DNA at 1 μg using Lipofectamine delivery system (Invitrogen, CA, USA) for 24 h. After selection by G418 antibiotic, cells were seeded into a 96-well plate at a density of 5 cells/ml to generate a single colony per well.

### Immunoprecipitation

Cell lysates (500 μg) were diluted in 1 ml cell lysis buffer containing 10 mM Tris-HCl pH 7.4, 140 mM NaCl, 3 mM MgCl_2_, 2 mM EDTA, 5 mM EGTA, 0.5% Triton X-100, and 4% protease inhibitor cocktail (Merck Biosciences) and incubated with rabbit anti-p19 CASP7 or mouse anti-XIAP antibody (2 μg of each), and rabbit anti-p17 CASP3 or mouse anti-XIAP antibody (2 μg of each) overnight at 4 °C, followed by precipitation with 20 μl protein A-agarose beads for 2 h at 4 °C. Immunoprecipitates were analyzed by SDS-PAGE/Western blotting using CCT-β, β-tubulin, XIAP, p19 CASP7, and p17 CASP3 antibodies. In addition, blots were stripped using a commercial kit (Millipore) and reprobed with anti-XIAP or anti-p19-CASP7, and anti-XIAP or anti-p17 CASP3 antibodies.

### Determination of caspase activities

The caspase activity assay was performed according to the manufacturer’s guidelines (BioVision, CA, USA). Briefly, cell lysates (200 μg) were diluted in 50 μl of cell lysis buffer (supplied by the kit). An equal volume of 2x reaction buffer (supplied by the kit) containing 10 mM DTT was added to the cell lysates. Subsequently, 50 μM of fluorescent dye-conjugated caspase substrates were individually added to the designated caspase activity assay. After 2-h incubation, free fluorescent dyes in the solution were read in a fluorimeter equipped with a 385 nm excitation filter and a 510 nm emission filter. Fold increases in caspase activity were determined by comparing these results with the level of the untreated control.

### MTT assay

Cells were plated in 100-µl medium per well in a 96-well plate and incubated at 37 °C under 5% CO_2_ overnight to allow the cells to attach to the wells. **643943**, STS, doxorubicin or paclitaxel dissolved in DMSO was added to each well to reach indicated concentrations when the growing area of cells achieved 70% of the wells. Mixed samples were incubated at 37 °C under 5% CO_2_ for 24 or 48 h to allow compounds to take effect. Ten µl MTT stock solution (5 mg/ml in DPBS) was added to each well. After thoroughly mixing with the medium, the mixture was then incubated at 37 °C under 5% CO_2_ for 4 h to allow the MTT to be metabolized. The medium was dumped off and formazan (MTT metabolic product) dissolved in DMSO (50 µl) was added per well. The absorbance at 490 nm was measured by using VMax ELISA Reader (Molecular Devices).

### PI-based flow cytometry analysis of the apoptotic cells

Cells were plated in 2-ml medium per well in a 6-well plate and incubated at 37 °C under 5% CO_2_ overnight to allow the cells to attach to the wells. **643943** dissolved in DMSO was added to wells to reach various concentrations (6.25, 12.5, 25, 50, and 100 µM) when 70% of each well was covered with cells. Mixed samples were incubated at 37 °C under 5% CO_2_ for 24 h to allow **643943** to take effect. The cells (6 × 10^5^) were collected to 15-ml tubes and 400 µl of 70% ice-cold alcohol (-20°C) was added to fix the cell membrane. After fixing for 15 min, the PI staining solution (0.1% BSA, 0.1% RNase A, and 20 ng/ml PI in PBS) was added and incubated for 30 min in the dark at room temperature (22–25 °C) to allow the PI to integrate with DNA. The samples were analyzed by the flow cytometer to assess DNA contents. The cells accumulated in the sub-G_0_ region were defined as apoptotic cells.

### In vitro activity and binding assays

Activity levels of 4.25 nM CASP7 without or with GST-linker-BIR2 (4.25 nM) plus 100 μM of **643943,** **119**, a SMAC peptide that binds to XIAP, or the mutant peptide that does not bind in the buffer containing 25 mM Tris, pH 7.5, 150 mM NaCl and 0.5% DMSO were measured using Ac-DEVD-AFC substrate. For the fluorescence experiments, the spectra of CASP7 (7 μM) in the absence or presence of increasing concentrations of **643943** or **119** (1, 3, 5, 10, and 15 μM) were scanned from 300-500 nm using a fluorimeter. The CASP7 concentrations were determined by Bradford method and calculated based on its MW of 70 kDa as a hetero-tetramer. For the BIAcore experiments, 2.5, 5 or 10 μM of **643943** was injected to mix with immobilized CASP7 and the SPR signals were recorded with time. The data were fit to yield the *K*_d_ for the binding of **643943** with CASP7. In competition experiments, GST-linker-BIR2 (0.14 μM) plus increasing concentrations of **643943** or **119** (0, 0.5, 10, 15, 20, 30, and 40 µM) were co-injected to mix with the immobilized CASP7.

### Animal models

All animal work was done in accordance with the protocol approved by the Institutional Animal Care and Use Committee of Academia Sinica and National Taiwan University. MCF-7 cells (5 × 10^5^) or MDA-MB-468 cells (5 × 10^5^) were suspended in 100 μl PBS containing 50% Matrigel (Corning) and subcutaneously inoculated into each 6-week-old immunodeficient NOD/SCID (ASID) mouse (National Laboratory Animal Center, RMRC13427). After the inoculation for 2 weeks, mice were randomly assigned to two groups (*n* = 6) that received vehicle control or **643943** (20 mg/kg) by intraperitoneal injection in 100 μl 30% solutol daily. Subcutaneous tumors were measured every 3 days using calipers, and their volumes were calculated using a standard formula (1/2 × wide × wide × length). Mice were sacrificed after 2 weeks post-injection with **643943**.

### TUNEL assay

In situ detection of apoptotic cells was conducted using the TUNEL assay kit according to the manufacturer’s protocol (Millipore). Briefly, sections were deparaffinized and quenched of endogenous peroxidase activity with 3% H_2_O_2_. Labeling was carried out by adding terminal deoxynucleotidyl transferase enzyme mix to the tissue sections, and the reaction was stopped by immersing slides in stop buffer. A diaminobenzidine peroxidase substrate kit (Vector Laboratories Inc.) was used to develop color following incubation with anti-digoxigenin conjugate. Subsequently, slides were stained with hematoxylin (Ventana) for 3 min at room temperature. Images were captured with an Olympus IX70 microscope using a ×40 objective.

## Supplementary information

Supplementary figures

Original Data

aj-checklist

## Data Availability

Data generated in this study are available upon request from the authors.
