# Supplementary material for: Blocking XIAP:CASP7-p19 selectively induces apoptosis of CASP3/DR malignancies by a novel reversible small molecule
Source: Cell Death Dis. 2025 Jun 18;16(1):459. doi: 10.1038/s41419-025-07774-y (PMC12177060; doi:10.1038/s41419-025-07774-y)
Supplement: Supplementary file 1 — Supplementary figures [file 41419_2025_7774_MOESM1_ESM.pdf]

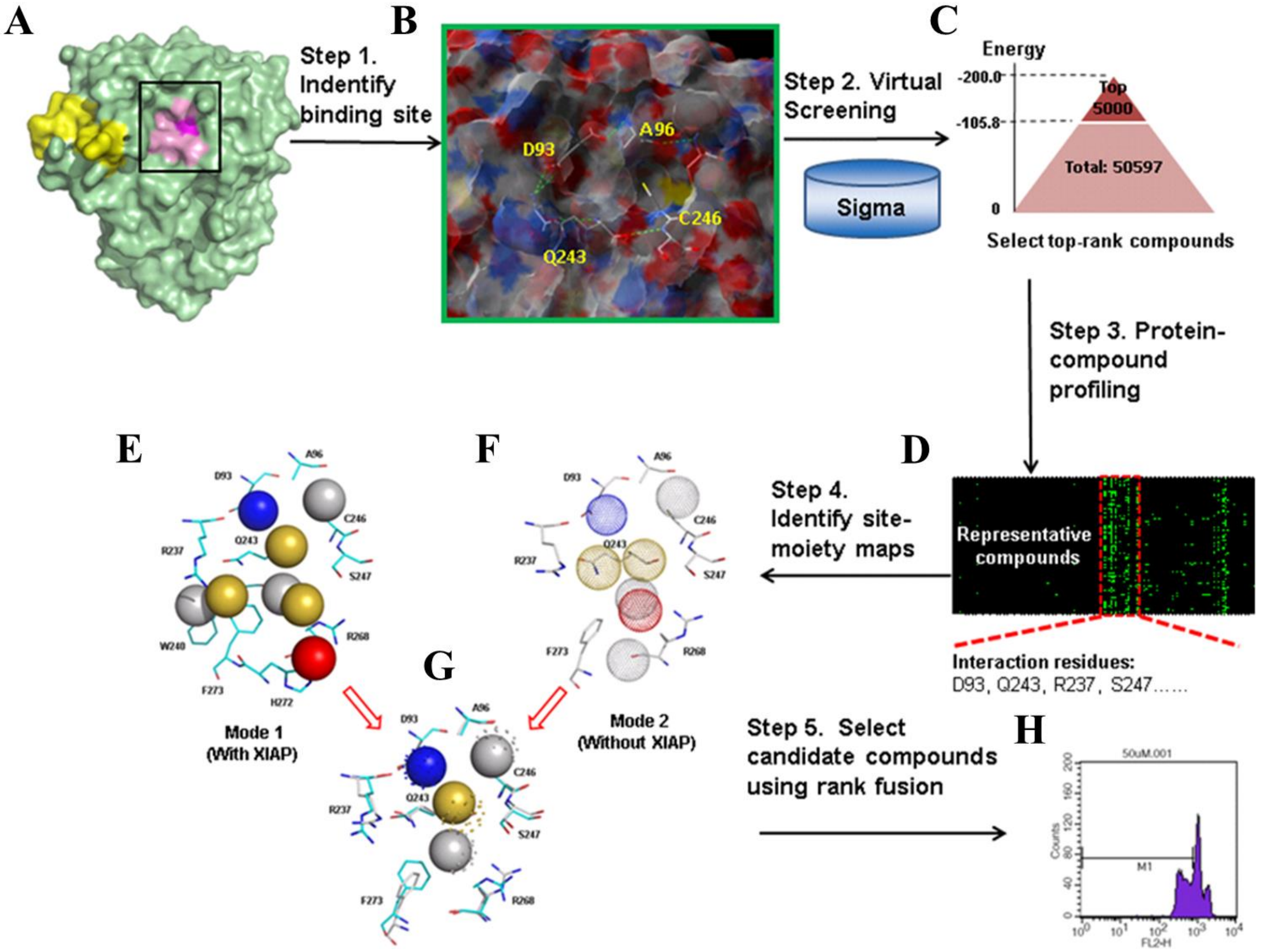

Supplementary Figure 1. Virtual Screening Strategy and Site-moiety Maps for Identifying the PPI Inhibitor. (**A**) The hot spot around Cys246 of CASP7 and (**B**) the site with four amino acids shown was used for virtual screening to identify the PPI inhibitor. (**C**) Compound library (Sigma-Aldrich) was screened using GEMDOCK and top-ranking 5000 compounds were selected for generating (**D**) residue-compound interaction profiles. Site-moiety map for CASP7 with XIAP (**E**) and that for free CASP7 (**F**) were derived based on conserved interactions between binding pockets and chemical moieties. (**G**) A common site-moiety-map was identified. (**H**) Compound candidates based on rank-based consensus scores combining energy-based and anchor-based ranks were selected for bioassay.

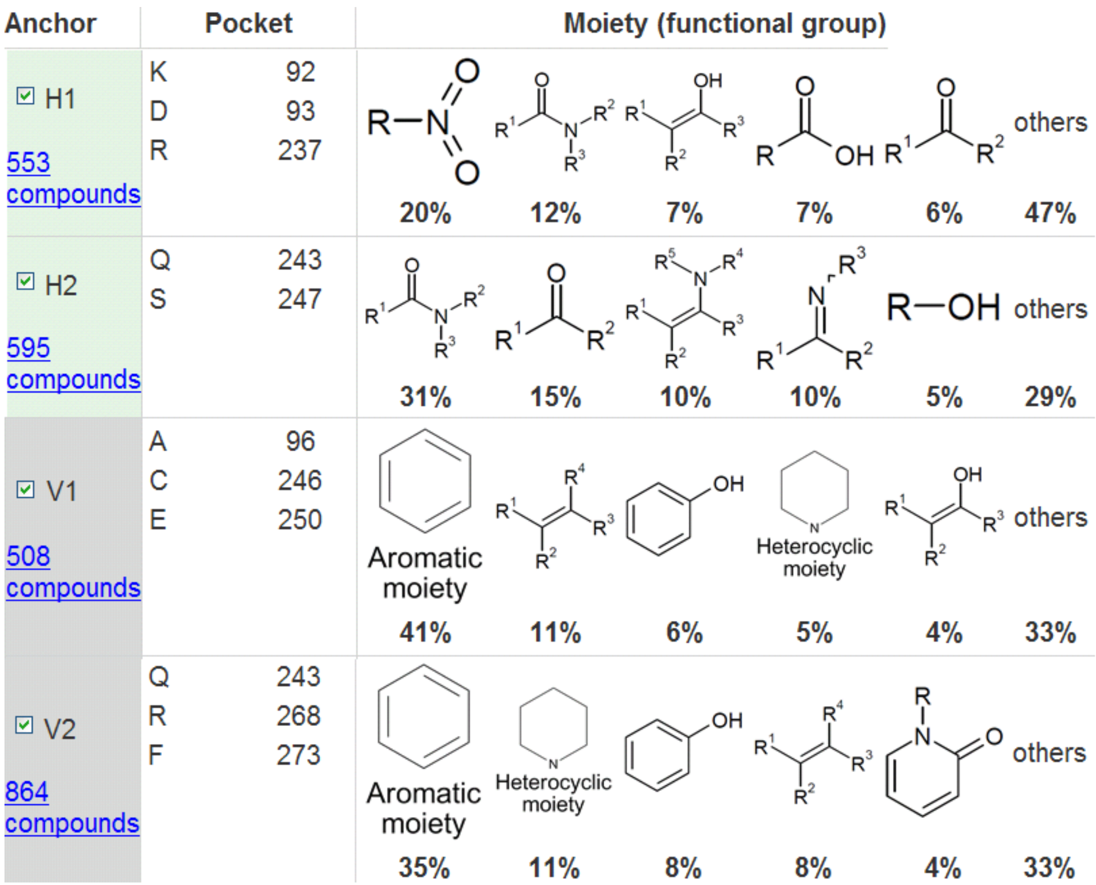

Supplementary Figure 2. The chemical moieties of common site-moiety-maps. The moiety compositions of four consensus anchors on the binding modes 1 (with linker-BIR2 of XIAP) and 2 (without XIAP) located at the **643943** binding site of CASP7. These results were produced by SiMMap.

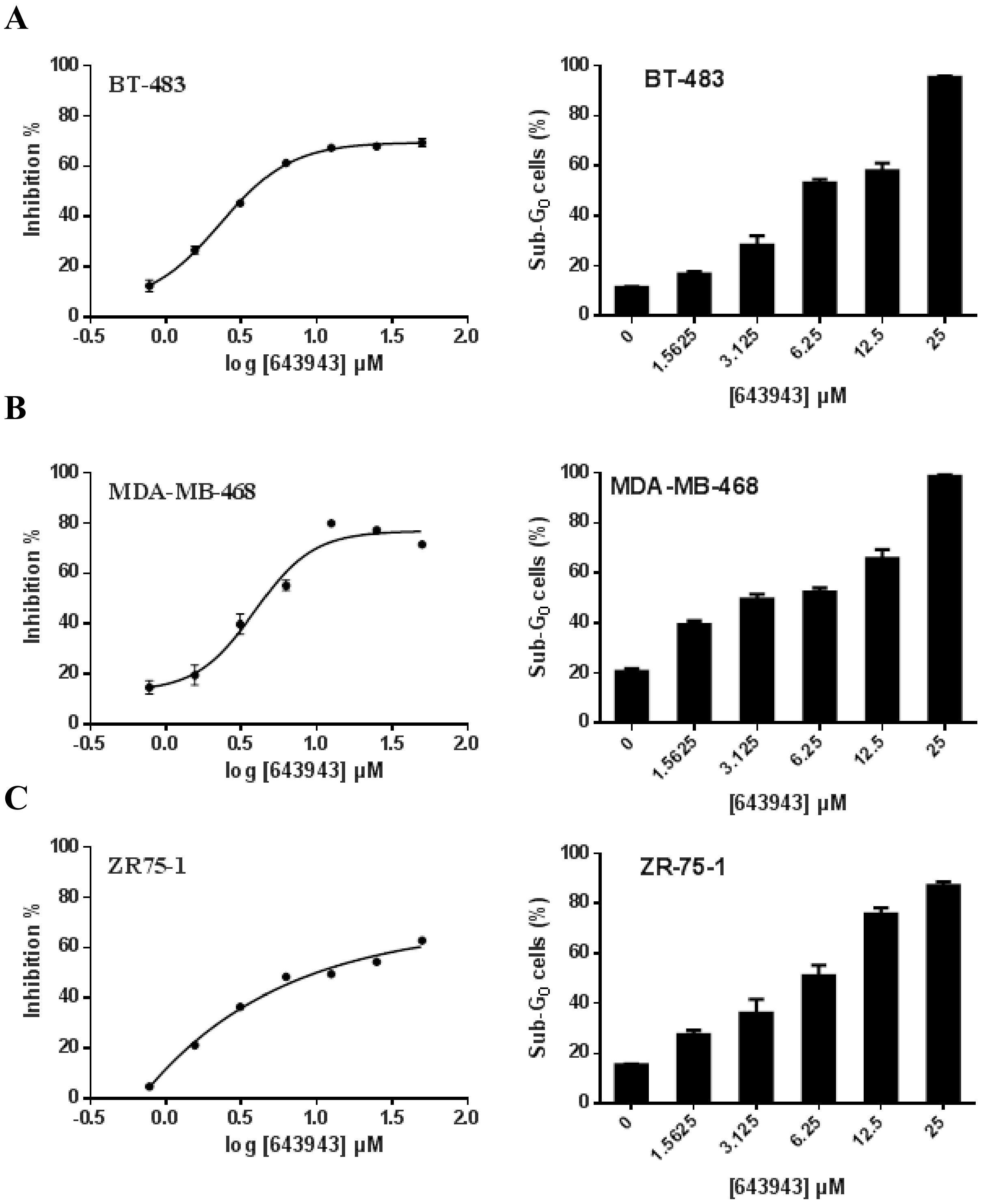

Supplementary Figure 3. The MTT assay and flow-cytometric assays for **643943** on the cell growth inhibition of BT-483 (**A**), MDA-MB-468 (**B**) and ZR-75-1 (**C**). Data were obtained from three independent experiments.
