# Supplementary material for: Blocking XIAP:CASP7-p19 selectively induces apoptosis of CASP3/DR malignancies by a novel reversible small molecule
Source: Cell Death Dis. 2025 Jun 18;16(1):459. doi: 10.1038/s41419-025-07774-y (PMC12177060; doi:10.1038/s41419-025-07774-y)
Supplement: Supplementary file 2 — Original Data [file 41419_2025_7774_MOESM2_ESM.pdf]

Original Data

A

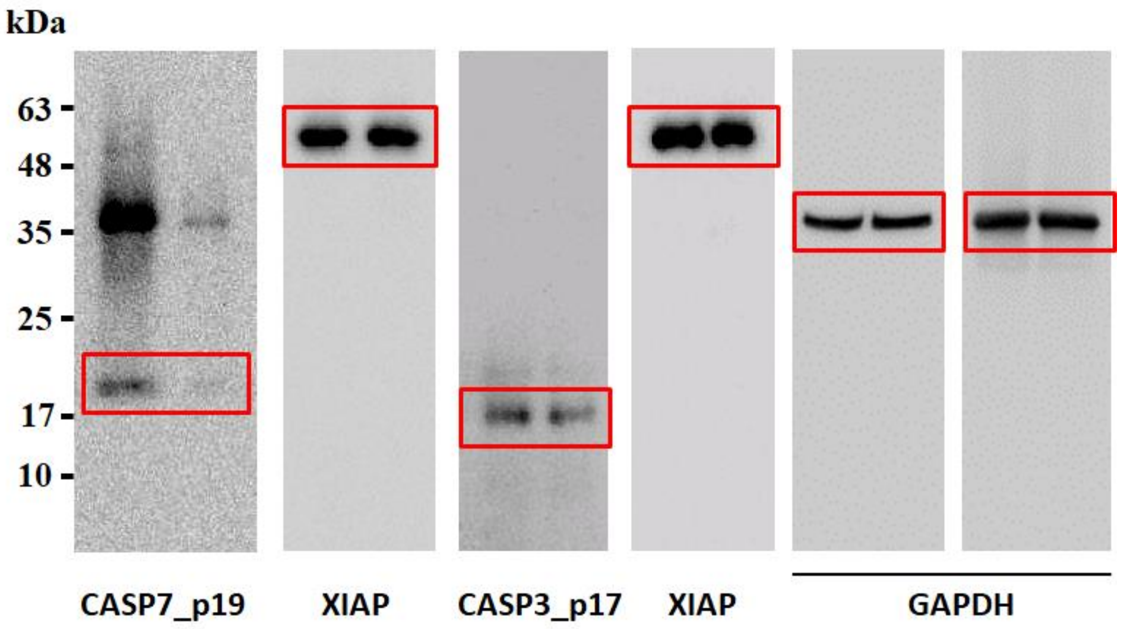

B

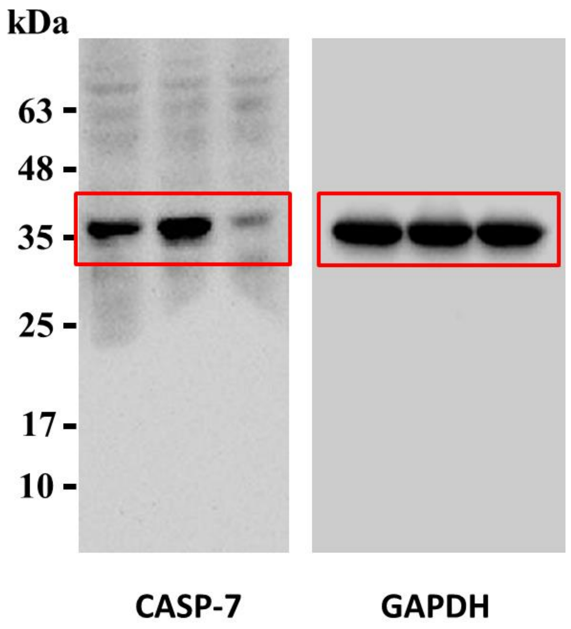

C

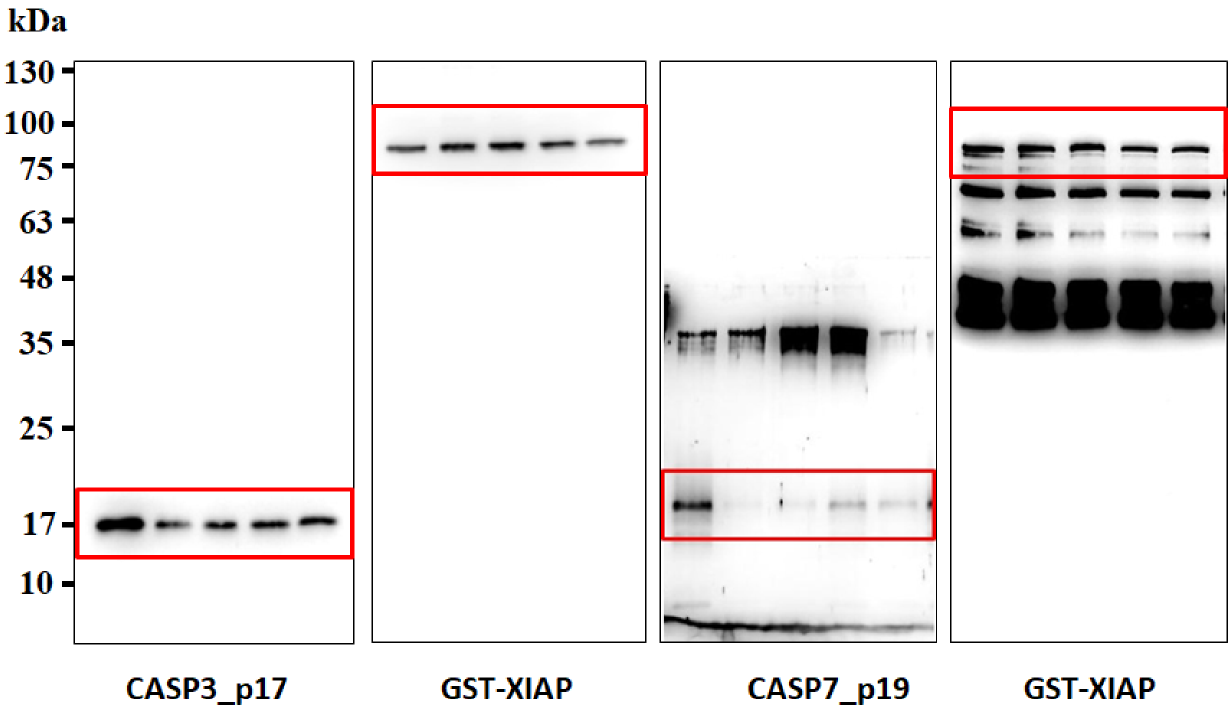
kDa
130
100
75
63
48
35
25
17
10
CASP3_p17
GST-XIAP
CASP7_p19
GST-XIAP

D

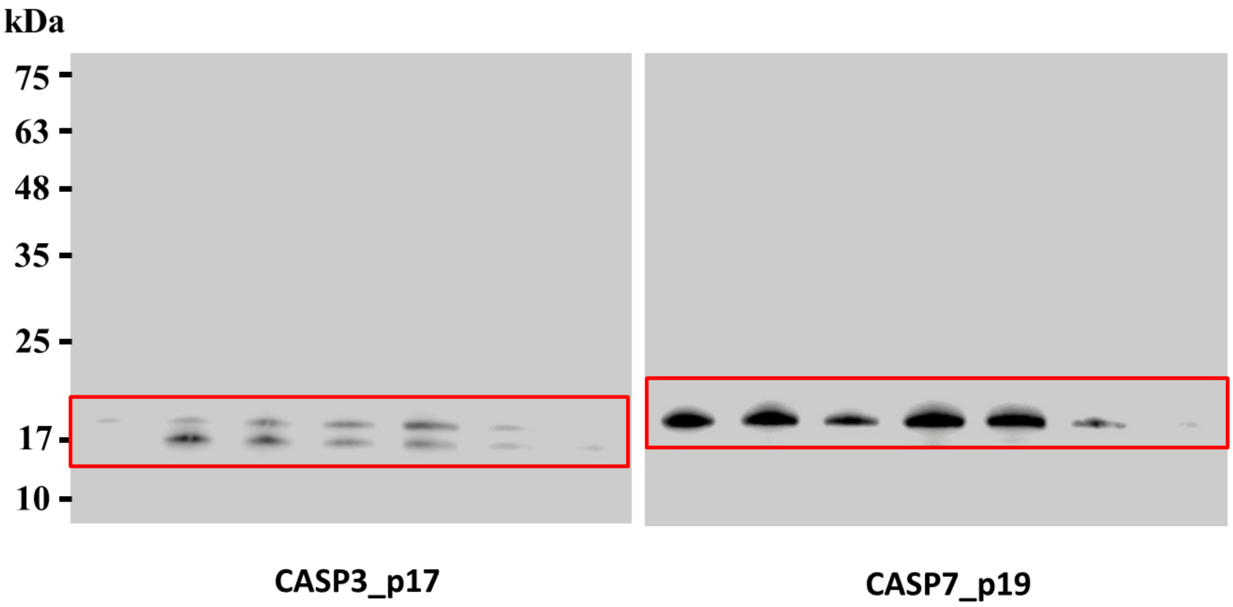
kDa
75
63
48
35
25
17
10
CASP3_p17
CASP7_p19

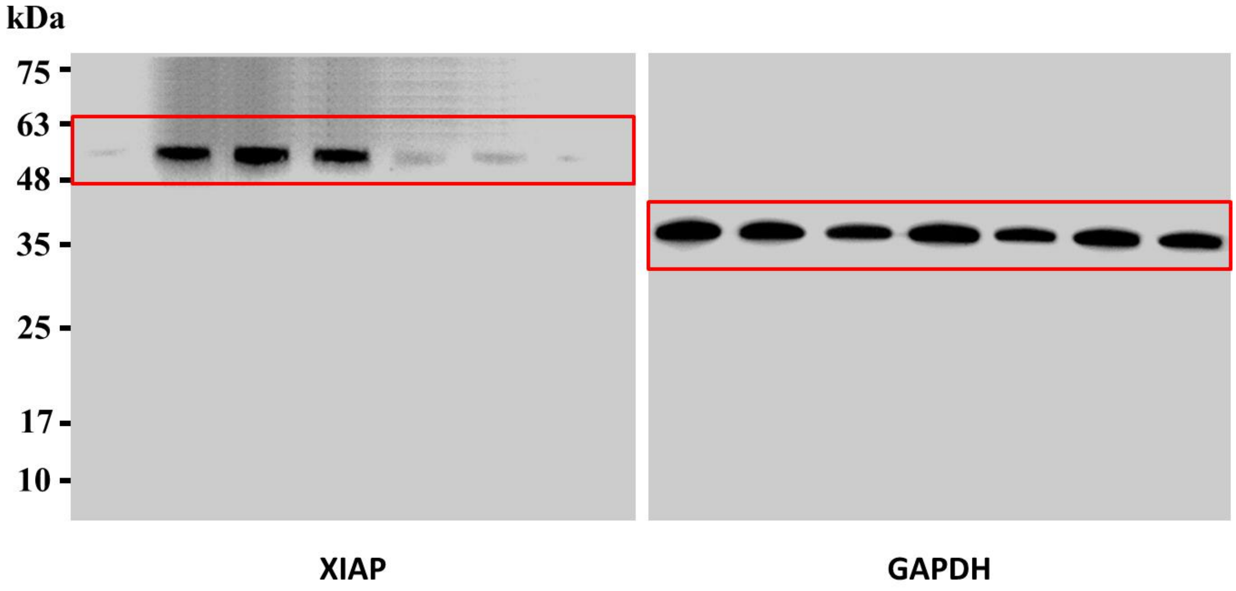
kDa
75
63
48
35
25
17
10
XIAP
GAPDH

E

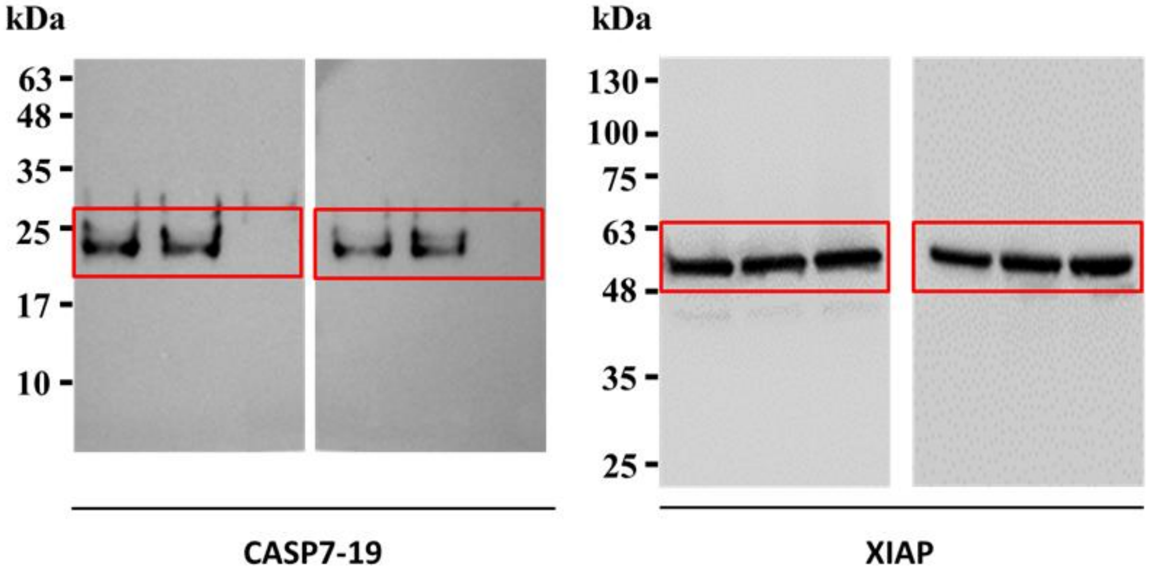
kDa
63
48
35
25
17
10
CASP7-19
kDa
130
100
75
63
48
35
25
XIAP

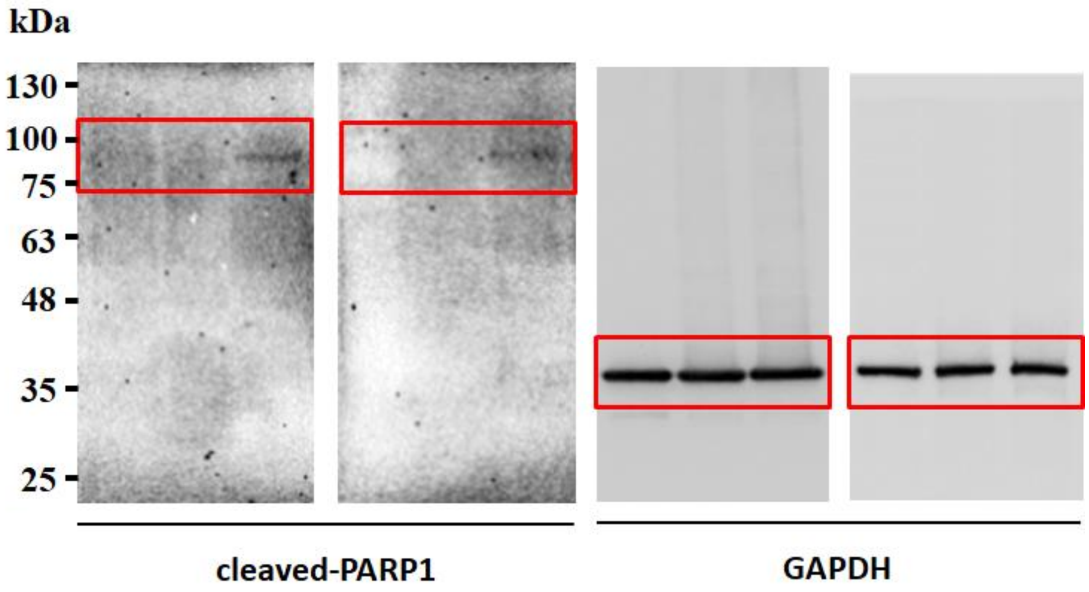
kDa
130
100
75
63
48
35
25
cleaved-PARP1
GAPDH

F

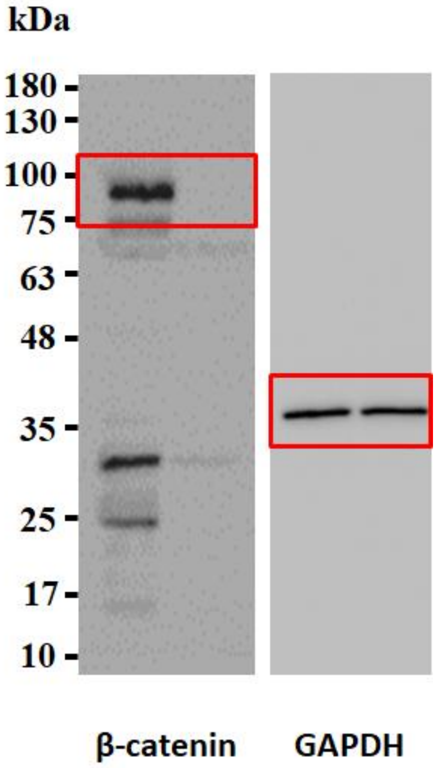
kDa
180
130
100
75
63
48
35
25
17
10
β-catenin
GAPDH

G

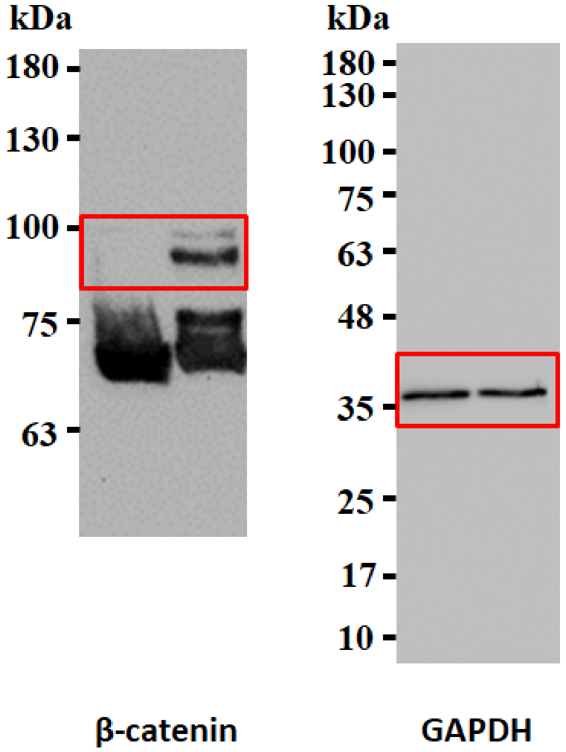
kDa
180
130
100
75
63
kDa
180
130
100
75
63
48
35
25
17
10
β-catenin
GAPDH

H

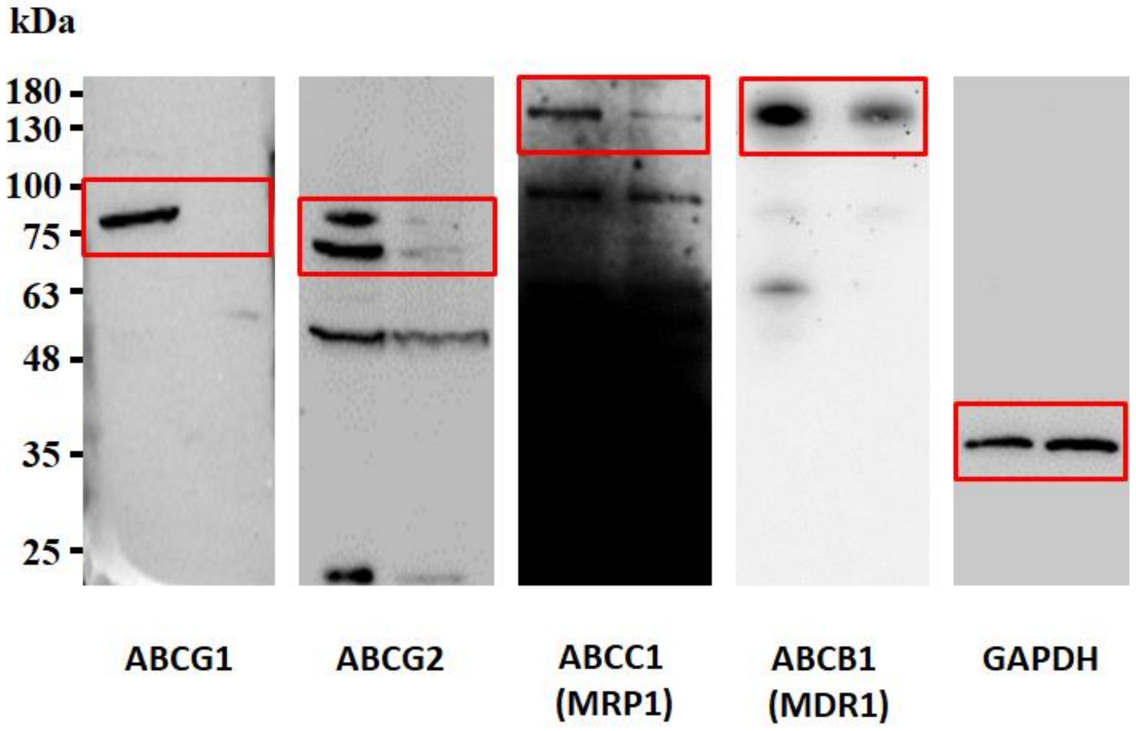

Full length original western blots. A Representative western blot analysis of CASP7_p19, XIAP, and CASP3_p17 (relative to Figure 2A). GAPDH was used as loading control. **B** Representative western blot analysis of CASP-7 in MCF-7 cells transfected without or with shRNA (relative to Figure 2G). GAPDH was used as loading control. **C** Representative western blot analysis of CASP3-p17, CASP7-p19, and GST-XIAP following pull down assays (relative to Figure 3A). **D** Representative western blot analysis of CASP3-p17, CASP7-p19, and XIAP in indicated cell lines (relative to Figure 5A). GAPDH was used as loading control. **E** Representative western blot analysis of CASP7_p19, XIAP, and cleaved PARP (relative to Figures 6A and 6B). GAPDH was used as loading control. **F** Representative western blot analysis of β-catenin in MCF-7/TR cells treated without or with **643943** (relative to Figure 6F). GAPDH was used as loading control. **G** Representative western blot analysis of β-catenin in MCF-7/TR cells pretreated without or with MPS following **643943** treatment (relative to Figure 6G). GAPDH was used as loading control. **H** Representative western blot analysis of ABCG1, ABCG2, ABCC1, and ABCB1 in 7TR cells treated without or with **643943** (relative to Figure 6H). GAPDH was used as loading control.
